# From Publication to Scientific Communication: The Expanding Role of Social Media in the Future of TJAR

**DOI:** 10.4274/TJAR.2026.262806

**Published:** 2026-08-28

**Authors:** Burhan Dost, Ceyda Özhan Çaparlar, Zekeriyya Alanoğlu

**Affiliations:** 1 Ondokuz Mayıs University Faculty of Medicine, Department of Anaesthesiology and Reanimation, Samsun, Türkiye; 2 University of Health Sciences Türkiye, Ankara Etlik City Hospital, Department of Anaesthesiology and Reanimation, Ankara, Türkiye; 3 Washington University in St. Louis, Faculty of Medicine, Department of Anaesthesiology, St. Louis, United States

In an era characterised by the rapid evolution of scientific knowledge and shifting patterns of access to scholarly information, social media have assumed an increasingly important role in academic publishing.^1^ As the influence of traditional print media continues to decline and digital platforms emerge as the predominant channels for disseminating scientific content, the judicious, ethical, and strategic use of social media by academic journals has transitioned from an optional consideration to an operational imperative.^2^ Given that many anaesthesiologists, researchers, and trainees now update their knowledge not only through traditional journal publications but also through social media platforms, (e.g., Instagram, LinkedIn, Facebook, and X), these platforms represent an important opportunity to reach wider and more diverse audiences. In addition to increasing the visibility of published articles, social media may facilitate scientific discussions, engage early-career researchers, and strengthen a journal’s national and international academic identity.

Importantly, the publication of an article should not be regarded as the endpoint of its scientific journey. Rather, it marks the beginning of a broader process of scientific communication that includes dissemination, interpretation, and meaningful engagement with the scientific community. Dissemination involves extending the reach of published research beyond conventional journal readership. Interpretation involves presenting complex findings in accessible, accurate, and contextually appropriate formats without compromising their scientific meaning. Engagement involves creating opportunities for readers, authors, reviewers, clinicians, and professional societies to interact with the research and with one another. Together, these processes can transform a published article from a static academic output into a dynamic resource for scientific communication.

In this context, social media can serve as an effective bridge between published evidence and clinical awareness, particularly in anaesthesiology, where emerging evidence frequently informs perioperative care and clinical decision-making. For the *Turkish Journal of Anaesthesiology and Reanimation *(TJAR), social media represents an important opportunity to strengthen its academic visibility and international reach. As a journal covering anaesthesiology, intensive care, pain medicine, perioperative medicine, regional anaesthesia, and patient safety, the TJAR has already begun using digital platforms to highlight high-quality research, promote educational content, and foster interaction among authors, readers, reviewers, editors, and professional societies. The journal’s recent Instagram metrics demonstrate sustained growth in audience reach, engagement, and visibility beyond its existing follower base (Figure 1), providing a practical example of how a structured social media strategy can extend the dissemination of scientific content beyond the publication of an article.^3^

The culture of scientific communication is currently undergoing a significant transformation. Traditionally, journals have disseminated newly published articles primarily through text-based announcements that direct readers to the original publication. However, digital audiences increasingly expect concise, visually organised, and platform-adapted scientific content that communicates the key message before encouraging further reading. In some cases, journals incorporate animation, narration, or even music to capture attention and improve engagement. This shift reflects a broader transition from simply announcing publications to actively communicating science (Figure 2). Within this evolving communication landscape, artificial intelligence (AI) has emerged as a valuable technology. AI-assisted tools can facilitate the preparation of visual abstracts, identify key messages, improve language clarity, adapt content for different audiences, and optimise the timing and format of social media posts.^4^ When integrated within a robust editorial workflow, these tools may improve the efficiency and consistency of scientific communication, while allowing editorial teams to focus on scientific quality and strategic communication. Nevertheless, AI should complement—not replace—editorial expertise. Human oversight remains essential to ensure scientific accuracy, contextual integrity, ethical compliance, transparency, and fidelity to the original publication.^5^ The objective is not to generate more content but to communicate high-quality science more clearly, responsibly, and effectively.

As anaesthesiology continues to evolve, journals must evolve alongside it. Moving beyond traditional publication models, social media has become an integral component of scientific communication, rather than simply a promotional tool. For TJAR, this transformation represents more than a communication strategy; it reflects a broader editorial vision of connecting research with clinicians, researchers, and the wider academic community. By combining scientific excellence, responsible digital communication, active community engagement, and thoughtful integration of AI-supported tools, the TJAR can further strengthen its position as a visible, accessible, and internationally engaged journal in the field of anaesthesiology.

## Figures and Tables

**Figure 1:**
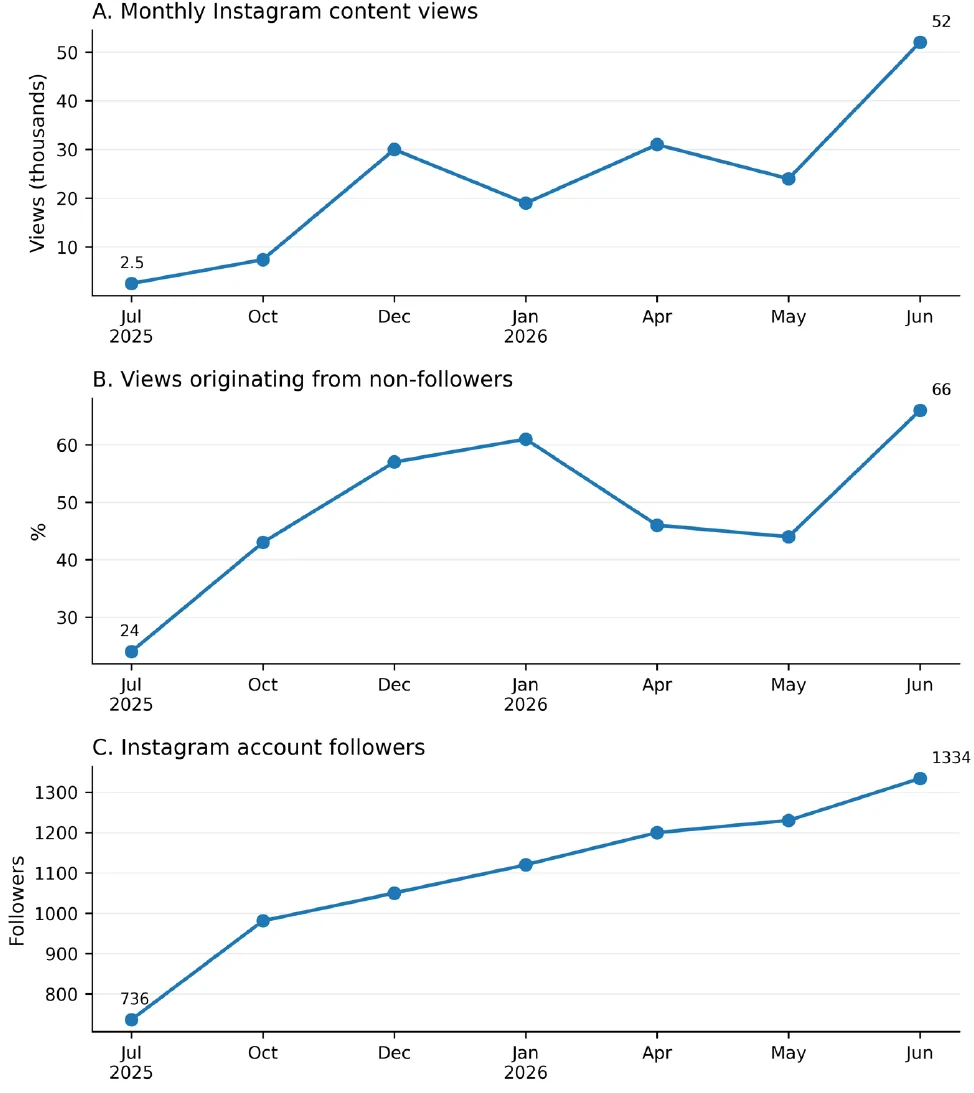
Instagram performance metrics of the *Turkish Journal of Anaesthesiology and Reanimation* (TJAR). (A) Monthly content views. (B) Percentage of views originating from non-followers. (C) Growth in Instagram account followers.

**Figure 2:**
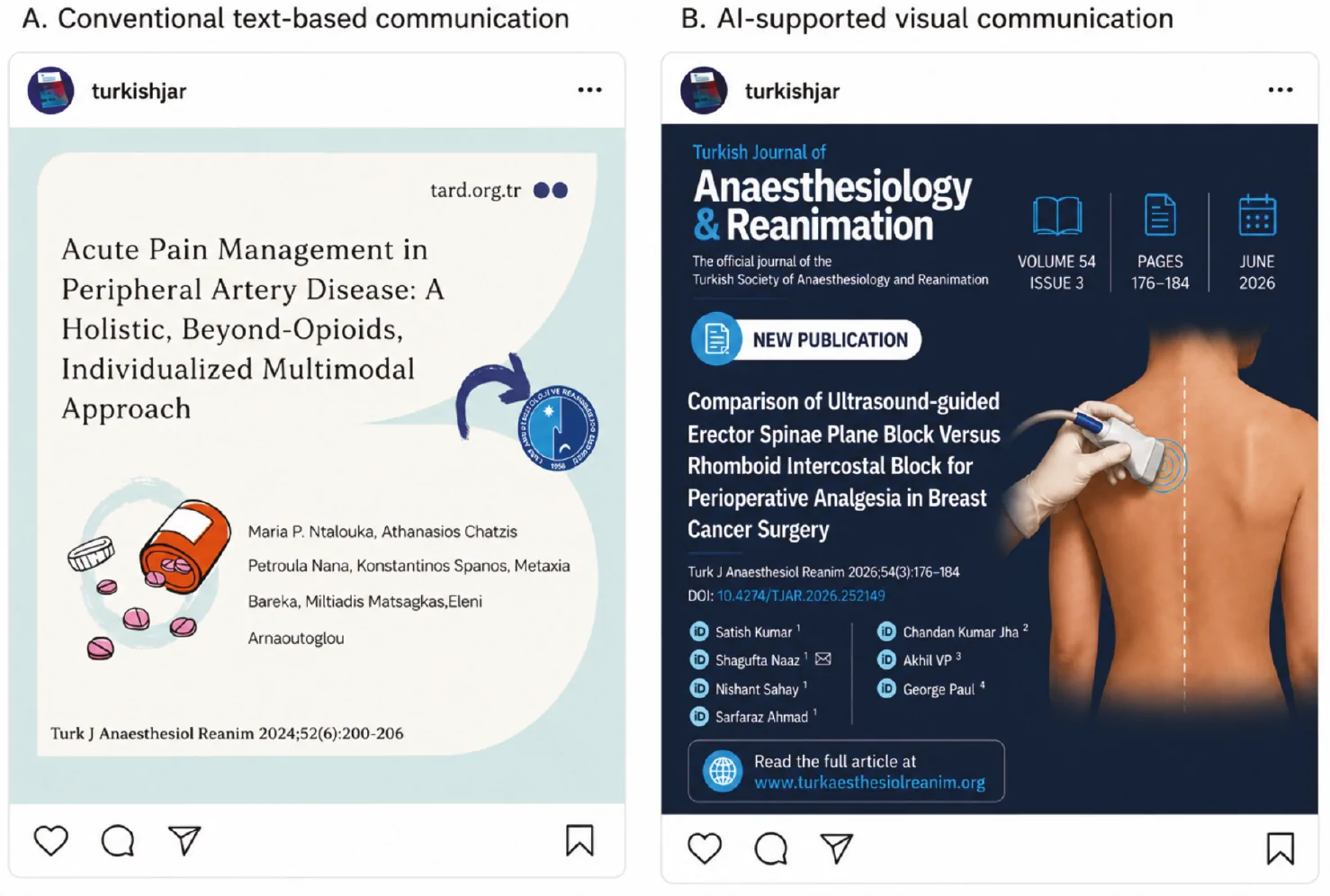
Evolution of social media communication in *Turkish Journal of Anaesthesiology and Reanimation *(TJAR). (A) Earlier text-oriented social media post primarily presenting bibliographic information. (B) Contemporary visual communication integrating structured layouts, medical illustration, and AI-supported design tools. AI, artificial intelligence.
